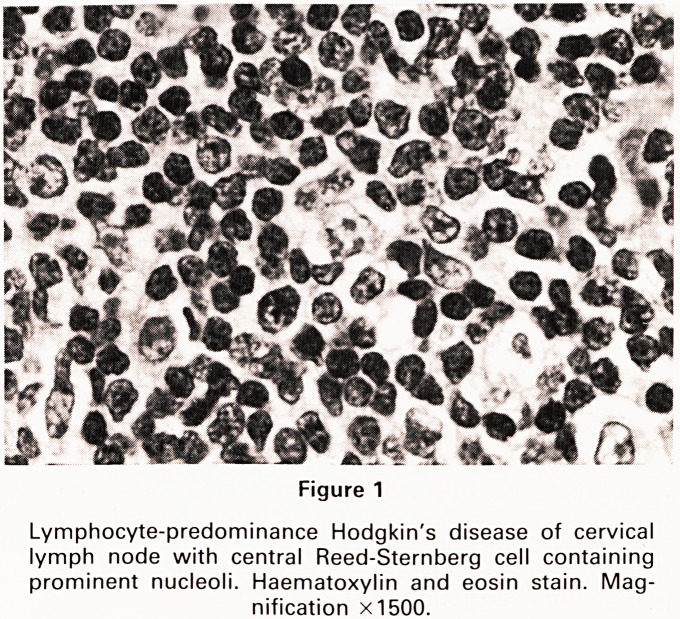# Phenytoin (Epanutin) Associated Hodgkin's Disease

**Published:** 1987-05

**Authors:** T. K. Daneshmend, J. D. Davies

**Affiliations:** University Department of Medicine; University Department of Pathology, Bristol Royal Infirmary, Bristol BS2 8HW

## Abstract

We report a case of phenytoin-associated Hodgkin's disease of lymphocyte predominance subtype, which developed after two years of phenytoin (Epanutin) treatment. Four other English cases of phenytoin-associated lmphoid malignancy are also reviewed.


Bristol Medico-Chirurgical Journal Volume 102 (ii) May 1987

### Phenytoin-(Epanutin)associated Hodgkin's disease

**T. K. Daneshmend, MD, MRCP**
University Department of Medicine.
**J. D. Davies, MD, FRCPath**
University Department of Pathology, Bristol Royal Infirmary, Bristol BS2 8HW.

### SUMMARY

We report a case of phenytoin-associated Hodgkin's disease of lymphocyte predominance subtype, which developed after two years of phenytoin (Epanutin) treatment. Four other English cases of phenytoin-associated lymphoid malignancy are also reviewed.

### INTRODUCTION

The pseudolymphoma syndrome is an unusual side effect of phenytoin treatment. This syndrome of lymphadenopathy, fever, rash, hepatosplenomegaly and eosinophilia usually resolves when phenytoin is stopped (1–3), though it may sometimes be fatal (4). On rare occasions phenytoin has also been associated with Hodgkin's disease and with non-Hodgkin lymphoma (5–7). The link between phenytoin and malignancy was first noted in the United States, from which most of the cases have been reported. Similar cases have also been reported from Australia (8–9), Israel (10), Spain (11) and Japan (12). However there have been no previous reports of phenytoin-associated Hodgkin's disease from the United Kingdom. We report such a case seen in Bristol, and review four other allied cases.

### CASE REPORT

In November 1977, a 28-year-old male steelworker with a six-month history of major epilepsy was treated with phenytoin 300 mg per day. Physical examination and all laboratory tests were normal before treatment. After 18 months of good control, his fits became more frequent. Serum phenytoin concentration was found to be subtherapeutic, so the dose was increased to 400 mg per day. In April 1980 he presented with a three week history of swelling on the left side of his neck. Examination showed enlarged lymph nodes along the border of the left sternomastoid and in the right axilla. There was no rash, fever, hepatosplenomegaly or eosinophilia. The full blood count, plasma viscosity and standard liver function tests were normal. Serum phenytoin was in the therapeutic range. A clinical diagnosis of phenytoin pseudolymphoma was made (though subsequently found to be incorrect), and carbamazepine substituted for phenytoin.

Histology of the excised cervical lymph nodes showed loss of nodal architecture and its replacement by nodules which contained scattered Reed-Sternberg cells of popcorn or polylobated (13) type in a background of small lymphocytes (Figure 1). At staging laparotomy no evidence of Hodgkin's disease was found below the diaphragm, though marked fibrosis was noted in excised para-aortic lymph nodes.

The patient remained free of fits and in May received radiotherapy to the mantle area and the para-aortic region. He has been well and free of Hodgkin's disease for over three years.

*Address for correspondence:* Dr T. K. Daneshmend, Department of Therapeutics, Floor C, South Block, University Hospital, Nottingham.

### OTHER POSSIBLE CASES

A computer search of the Adverse Reactions Register of the Committee on Safety of Medicines yielded four other possible cases of phenytoin-associated lymphoid malignancy (Committee on Safety of Medicines—personal communication). The brief details are given in Table 1. Unfortunately the histology from these other cases was not available to us for review.

### DISCUSSION

Hodgkin's disease is a not uncommon malignancy and phenytoin is a widely prescribed drug. Therefore, it is possible that the present case represents a chance association. There are no histological features which are specifically characteristic of phenytoin-associated lymphoid malignancies. Thus a direct relationship between drug and malignancy cannot be proven in the present case, or in those reported previously. However, phenytoin has been associated with a ten-fold increase in the incidence of malignant lymphoma (6), and a case-control study found a four-fold increase in phenytoin-associated malignant lymphoma (7).

**Figure 1**

Lymphocyte-predominance Hodgkin's disease of cervical lymph node with central Reed-Sternberg cell containing prominent nucleoli. Haematoxylin and eosin stain. Magnification ×1500.

**Table 1**

**Patients with phenytoin associated lymphoid malignancy reported to the Committee on Safety of Medicines**

| *CSM No.* | *Age (y)/Sex* | | *Duration of phenytoin* | *Site/Histological diagnosis* |
|---|---|---|---|---|
| 110477 | 77 | F | 6 years | Mesenteric nodes/follicular lymphoma |
| 109944 | 56 | F | 20 years | Cervical nodes/lymphoma |
| 108131 (present case) | 31 | M | 2.5 years | Cervical nodes/Hodgkin's disease |
| 97696 | 34 | F | 15 years | Not stated/Hodgkin's disease |
| 28943 | 47 | M | 14 years | Spine/malignant lymphoma |

Four types of histological lymph node change have been recognised in association with phenytoin (14). The first, reactive hyperplasia, is readily recognised on account of the preservation of nodal architecture, although the follicles may be atrophic. The second type of change is the pseudolymphomatous reaction. This resembles angioimmunoblastic lymphadenopathy, and may cause more diagnostic difficulty in view of the distortion of nodal architecture. Numerous immunoblasts, cells resembling Reed-Sternberg cells, focal necrosis often accompanied by vasculitis (15), and occasionally, conspicuous eosinophilia characterises this lymph node reaction, which regresses after withdrawal of phenytoin. In a third type of reaction, 'Pseudo-pseudolymphoma', the nodes become smaller after stopping phenytoin, but subsequently enlarge with the development of genuine malignant lymphoma. The fourth nodal pathology associated with phenytoin is malignant lymphoma. Most such cases have been of Hodgkin's disease, usually of mixed cellularity, but non-Hogdkin's lymphomas have also been reported (8).

Our case, which lacked reactive features, was clearly an example of Hodgkin's disease. Unlike the majority of such lymphomas seen in association with phenytoin, it was of the nodular lymphocyte-predominance subtype. It is important to note that features of the phenytoin pseudolymphoma syndrome, such as skin rashes, fever, gum hypertrophy and blood eosinophilia, were absent in the patient reported here and in those with malignant lymphoma reported earlier.

Lymphadenopathy with drugs other than phenytoin has been noted with carbamazepine (16), captopril (17), dapsone (18), primidone (C.S.M—personal communication), and excessive vitamin A intake (19). Lymphocytic lymphoma has been associated with dantrolene, a hydantoin similar to phenytoin (20). Lymphoma with drugs other than phenytoin is extremely uncommon.

The mechanism by which phenytoin causes lymphoid change and malignancy remains unknown. In animals, phenytoin behaves as a hapten, rather than a mitogen, and apparently renders the membranes of lymphoreticular cells antigenic for autologous T lymphocytes (21). It may also cause a failure of normal lymphoproliferative control mechanisms (21). In man, phenytoin depresses cellular and humoral immunity, though there is wide variation between individuals (22, 23).

We should like to stress that true malignancy in association with phenytoin is extremely rare, and restricted to the lymphoreticular system. We should therefore not wish to curtail the clinical use of phenytoin, except perhaps in patients with Hodgkin's disease or other lymphoma who are in remission or undergoing active treatment. A careful drug history, with specific questioning regarding phenytoin, should be sought from patients with lymphoid malignancy, and may yield further examples of the association presented here.

**ACKNOWLEDGEMENTS**

We thank Dr D R Coles, Physician, Bristol Royal Infirmary, for allowing us to report this case. Our thanks also to Dr J C P Weber. Senior Medical Officer, Committee on Safety of Medicines, London.

**REFERENCES**

1. SALTZSTEIN, S. L. and ACKERMAN, L. V. (1959) Lymphadenopathy induced by anticonvulsant drugs and mimicking clinically and pathologically malignant lymphomas. *Cancer*, **12**, 164–182.

2. ROSENFELD, S., SWILLER, A. I.. SHENOY, Y. M. V. and MORRISON, A. N. (1961) Syndrome simulating lymphosarcoma induced by diphenylhydantoin sodium. *JAMA*, **176**, 491–493.

3. GAMS, R. A., NEAL, J. A., and CONRAD, F. G. (1968) Hydantoin induced pseudo-pseudo-lymphoma. *Ann.Intern.Med.*, **69**, 557–568.

4. McCARTHY, L. J. (1979) Fatal bening pheytoin lymphadenopathy. *Arch.Intern.Med.*, **139**, 367–368.

5. HYMAN, G. A. and SOMMERS, S. C. (1966) The development of Hodgkin's disease and lymphoma during anticonvulsant therapy. *Blood*, **28**, 416–426.

6. ANTHONY, J. J. (1970) Malignant lymphoma associated with hydantoin drugs. *Arch.Neurol.*, **22**, 450–454.

7. LI, F. P., WILLARD, D. R., GOODMAN, R. and VAWTER G. (1975) Malignant lymphoma after diphenylhydatoin (Dilantin) therapy. *Cancer*, **36**, 1359–1362.

8. BROWN, J. M. (1971) Drug-associated lymphadenopathies with special reference to the Reed-Sternberg cell. *Med. J.Aust.*, **1**, 375–378.

9. SORRELL, T. C. and FORBES, I. J. (1975) Phenytoin sensitivity in a case of phenytoin associated Hodgkin's disease. *Aust.N.Z.J.Med.*, **5**,144–147.

10. MATZNER, Y. and POLLIACK, A. (1978) Lymphoproliferaive disorders in four patients receiving chronic diphenylhydantoin therapy: etiologic correlation or chance association. *Israel J.Med.Sci.*, **8**, 865–869.

11. CREIXENTI, J. B., PORTA, F. S., XARNAN, S. N., MARTIN, E. S. and SAN MIGUEL, J. G. (1980) Asociacion de enfermedad de Hodgkine hidantoinas. *Medicina Clinica (Barcelona)*, **75**, 24–26.

12. ISOBE, T., HORIMATSU, T., FUJITA, T., MIYAZAKI, K. and SUGIYAMA, T. (1980) Adult T cell lymphoma following diphenylhydantoin therapy. *Arch.Haematol.Jpn.*, **43**, 711–714.

13. WRIGHT, D. H. and ISAACSON, P. G. (1983) *Biopsy Pathology of the Lymphoretcular System.* London, Chapman & Hall, pp 93–96.

14. SYMMERS, W. StC. (1978) *Systemic Pathology.* 2nd Edn. Edinburgh, Churchill Livingstone, 1978. Vol 2, pp 697–701.

15. DAVIES, J. D. (1985) Vascular disorders in Stansfeld, A. G. (ed), *Lymph Node Biopsy Interpretation.* Churchill Livingsone, Edinburgh, pp 142–158.

16. LEWIS, I. J. and ROSENBLOOM, L. (1982) Glandular fever-like syndrome, pulmonary eosinophilia and asthma associated with carbamazepine. *Postgrad.Med.J.*, **58**, 100–101.

*(continued on page 50)*

### Phenytoin-(Epanutin) associated Hodgkin's disease (Continued from page 36)

17. ABERG, H., MORLIN, C. and FIRTHZ, G. (1981) Captopril-associated lymphadenopathy. *Br.Med.J.*, **283**, 1297–1298.
18. TOMECKI, K. J. and CATALANO, C. J. (1981) Dapsone hypdersensitivity, the sulfone syndrome revisited. *Arch.Dermatol.*, **117**, 38–39.
19. CLARK, F. (1981) Disorders of metabolism II. Ch 15 in Davies, D. M. (ed), *Textbook of Adverse Drug Reactions.* 2nd Edn. Oxford, Oxford, pp 330–405.
20. WAN, H. H. and TUCKER, J. S. (1980) Dantrolene and lymphocytic lymphoma. *Postgrad.Med.J.*, **56**, 261–262.
21. GLEICHMANN, E., VAN ELVEN, F. and GLEICHMANN H. (1979) Immunoblastic lymphadenopathy, systemic lupus erythematosis and related disorders. *Amm.J.CLIN.Pathol.*, **72**, 708–723.
22. SORRELL, T. C. and FORBES, I. J. (1975) Depression of immune competence by phenytoin and carbamazepine. *Clin.Exp.Immunol.*, **20**, 273–285.
23. BARDANA, E. J., GABOUREL J. D., DAVIES, G. H. and CRAIG, S. (1983) Effect of phenytoin on man's immunity. *Am.J.Med.*, **74**, 289–296.

## Figures and Tables

**Figure 1 f1:**